# Surgical Treatment of Sporadic Pancreatic Neuroendocrine Tumors: A State of the Art Review

**DOI:** 10.1100/2012/357475

**Published:** 2012-12-10

**Authors:** Sven-Petter Haugvik, Knut Jørgen Labori, Bjørn Edwin, Øystein Mathisen, Ivar Prydz Gladhaug

**Affiliations:** ^1^Department of Hepato-Pancreato-Biliary Surgery, Rikshospitalet, Oslo University Hospital, Sognsvannsveien 20, 0372 Oslo, Norway; ^2^Institute of Clinical Medicine, University of Oslo, Kirkeveien 166, 0450 Oslo, Norway; ^3^Interventional Centre, Rikshospitalet, Oslo University Hospital, Sognsvannsveien 20, 0372 Oslo, Norway

## Abstract

Pancreatic neuroendocrine tumors (PNETs) are rare neoplasms. They are clinically diverse and divided into functioning and nonfunctioning disease, depending on their ability to produce symptoms due to hormone production. Surgical resection is the only curative treatment and remains the cornerstone therapy for this patient group, even in patients with advanced disease. Over the last decade there has been a noticeable trend towards more aggressive surgery as well as more minimally invasive surgery in patients with PNETs. This has resulted in improved long-term survival in patients with locally advanced and metastatic disease treated aggressively, as well as shorter hospital stays and comparable long-term outcomes in patients with limited disease treated minimally invasively. There are still controversies related to issues of surgical treatment of PNETs, such as to what extent enucleation, lymph node sampling, and vascular reconstruction are beneficial for the oncologic outcome. Histopathologic tumor classification is of high clinical importance for treatment planning and prognostic evaluation of patients with PNETs. A constant challenge, which relates to the treatment of PNETs, is the lack of an internationally accepted histopathological classification system. This paper reviews current issues on the surgical treatment of sporadic PNETs with specific focus on surgical approaches and tumor classification.

## 1. Epidemiology

Pancreatic neuroendocrine tumors (PNETs) are rare and account for about 1-2% of all pancreatic neoplasms [1, 2]. The incidence has increased during the last decades to 4-5 per 100,000 in the general population [3–5]. Autopsy studies have shown that PNETs can be identified in as many as 10% of the population, suggesting that many carry asymptomatic disease [6]. Ten to 15% of all PNETs are part of familial syndromes such as multiple endocrine neoplasia type 1, von Hippel-Lindau, neurofibromatosis and tuberous sclerosis [3], which will not be reviewed further in this paper. The tumorigenesis and molecular pathogenesis of PNETs remain poorly understood. 

## 2. Clinical Presentation

PNETs are clinically diverse and divided into functioning and nonfunctioning disease, dependent on their ability to produce symptoms due to hormone production [7]. The distinction between nonfunctioning and functioning PNETs is based on immunohistochemistry of tumor tissue in addition to clinical symptoms. Thirty to 50% of all PNETs are nonfunctioning [8, 9]. Since nonfunctioning tumors do not cause hormone-dependent symptoms, they are often detected incidentally or through symptoms related to mass effect resulting from local or distant tumor progression [10]. Common symptoms of nonfunctioning PNETs are abdominal pain, nausea and/or vomiting, fatigue, obstructive jaundice, and abdominal mass [11, 12]. Patients with functioning PNETs, such as insulinoma and gastrinoma, often present with characteristic symptoms dependent on the hormones produced. However, the clinical relevance of the distinction between functioning and nonfunctioning PNETs has recently been questioned as the treatment of these tumors follow the same general principles [13]. 

## 3. Classification

Classification systems enable patient risk stratifications and directly impact clinical decision making [16]. PNETs are generally classified according to their tumor-node-metastasis (TNM) pattern, defined by TNM staging systems, and grading, defined by the WHO 2010 classification [4, 17]. The latter is based on the tumor antigen and cell proliferation marker Ki-67. A Ki-67 of below 2% corresponds to a neuroendocrine tumor (NET) G1, a Ki-67 of 2–20% corresponds to a NET G2, whereas a Ki-67 above 20% corresponds to a neuroendocrine carcinoma (NEC) G3 [17]. Beside the generally accepted grading system, there are currently two TNM staging systems that are applied for staging of PNET. One system was proposed by the International Union for Cancer Control, American Joint Cancer Committee and the World Health Organization (UICC/AJCC/WHO), and is widely used in the North American region, while the other system was proposed by the European Neuroendocrine Tumor Society (ENETS) and is predominant in the European region [14, 15, 18]. Several studies have demonstrated the usefulness of the ENETS TNM staging system [14, 19–22] and in a recent study, Rindi et al. found that the ENETS TNM staging system is superior to the UICC/AJCC/WHO 2010 TNM staging system in terms of prognostic stratification for patients with PNETs [14]. The ENETS TNM staging system is shown in Table 1. The final classification of PNETs is based on histopathological examination. The histology report should include a minimum set of criteria, including (1) a macroscopic description of the surgical specimen with exact anatomical site, margins distance, and size of the lesion, (2) a microscopic description with supporting immunohistochemistry, mitotic count, Ki-67 index, node-, and margin status, and (3) diagnosis with distinction between NET and NEC, grade, and TNM stage (Table 2). 

## 4. Surgery

Surgical resection is the only curative treatment for patients with PNETs and remains the cornerstone therapy [11, 23–26], even in patients with advanced disease. The goals for surgical resection are cure, relief from functioning tumors [27], or relief from nonfunctioning tumors causing symptoms related to mass effect (biliary obstruction, gastric outlet obstruction, abdominal pain, gastrointestinal hemorrhage). Resectability rates up to 65% have been reported [28]. However, a substantial portion of patients with PNETs initially present with advanced disease, which cannot be radically resected. 

### 4.1. Surgical Approaches

#### 4.1.1. Functioning Disease

Functioning PNETs primarily include insulinomas and gastrinomas, with an incidence of 70–80% and 20–25% of all PNETs, and an incidence of malignancy of <10% and 50–60%, respectively [9].

Insulinomas are generally solitary, benign, and curable with surgery [9, 28, 29]. Recurrence after resection occurs in about 3% [30, 31]. The procedures of choice are enucleation for small and isolated insulinomas and partial pancreatectomy for large and potentially malignant insulinomas [32, 33]. Beside enucleation, middle pancreatectomy is an alternative parenchyma-sparing technique for this tumor entity [34]. Also, laparoscopic management of insulinoma in the body and tail of the pancreas, with distal pancreatectomy or enucleation, is feasible and safe [35]. In the case of occult insulinoma, blind distal pancreatectomy should be avoided [36]. However, explorative surgery with intraoperative ultrasound may be indicated in cases where preoperative diagnostics could not reveal any pancreatic lesions, as this is an excellent method for identifying occult insulinoma [37].

Gastrinoma is associated with gastric ulcerations due to overproduction of gastrin [38]. The clinical presentation of gastrinoma is referred to as Zollinger-Ellison syndrome. With the introduction of proton pump inhibitors, which prevent ulcer formation, surgery changed from being symptomatic to curative treatment in patients with Zollinger-Ellison syndrome [28]. All patients with Zollinger-Ellison syndrome without multiple neuroendocrine neoplasia type 1 or metastatic disease should be offered surgical exploration for possible cure [39]. Routine use of duodenotomy in cases of pancreatic gastrinoma increases short- and long-term cure rates due to a higher detection rate of duodenal gastrinomas, as multiple gastrinomas are relatively common [40].

The incidences of other functioning PNETs, such as vasoactive intestinal peptide-producing tumors (VIPoma), glucagonoma, and somatostatinoma, are very low. These patients should undergo tumor resection to correct the severe hormonally caused metabolic derangements [28]. 

#### 4.1.2. Nonfunctioning Disease

Nonfunctioning PNETs represent 30–50% of all PNETs and malignancy occurs in 60–90% [8, 9, 41]. Even though curative surgery is rare in patients with nonfunctioning PNETs, long-term survival can be achieved in many patients [12]. There is a strict correlation between tumor size and malignancy in these tumors [42]. Tumors larger than 2 cm have an increased risk of malignancy [43]. Solitary benign nonfunctioning PNETs can be removed by enucleation or spleen- or duodenum-preserving techniques in most cases [8]. 

#### 4.1.3. Neuroendocrine Carcinoma

Neuroendocrine carcinomas (NECs) are defined as neuroendocrine tumors with a Ki-67 index above 20%, according to the WHO 2010 classification [17]. Such tumors are highly malignant and typically invade adjacent structures or metastasize before the diagnosis is made [44]. NECs of the pancreas are very rare and account for only about 2-3% of all PNETs [45–47]. The outcome is generally poor and most patients die within five years after diagnosis [44, 48]. However, curative resections have been reported in single cases [45]. Therefore, radical surgery should be attempted in localized disease [49, 50], while surgery in metastatic disease is not recommended [48]. 

#### 4.1.4. Locally Advanced Disease

Locally advanced disease extends beyond the limits of the pancreas directly into surrounding organs or tissue, involves regional lymph nodes, or fulfills both of these criteria [5]. As many PNETs are nonfunctioning and slow-growing, a large proportion of these present with locally advanced disease. Resection for locally advanced PNETs is in general technically feasible and can result in favorable disease-free and overall survival in selected patients [51]. However, most patients will develop recurrence [52]. When not operated, patients with locally advanced PNETs may suffer from complications related to local mass effect and infiltrative growth, including gastrointestinal bleeding, vascular/intestinal/biliary obstruction, and occlusion of the superior mesenteric (SMV) or portal vein (PV) [53]. Hill et al. found that resection of the primary tumor in patients with PNETs is associated with improved survival across all stages of disease [54]. Based on this, surgery of locally advanced PNET without metastasis should be attempted. Interestingly, R1 resections of PNET are not associated with a worse overall survival compared to R0 resections [21, 55]. 

#### 4.1.5. Metastatic Disease

PNETs commonly metastasize to the liver. This is especially true for nonfunctioning tumors as these are generally diagnosed at a late stage. In selected patients, resection of the primary PNET in the setting of unresectable but limited hepatic metastases may be indicated [56–58] as this may prolong survival [59–61]. As mentioned earlier, it has been shown that resection of the primary tumor in patients with PNETs is associated with improved survival across all stages of disease [54]. However, there is currently no clear answer to when and whether resection of the primary tumor should be performed in metastatic disease [62]. 

Surgical resection with curative intent or palliative debulking of more than 90% of liver metastases from nonfunctioning PNETs provides favorable oncologic outcomes, despite a high recurrence rate [63–65]. Patients with metastatic disease in the liver may profit from liver resection with long-term palliation and possibly cure in one-third of the patients [66]. Number, size, and localization of tumor sites seem less important than performing a complete resection of metastatic tissue from PNETs [67]. Patients with hormonally active liver metastases without prior extrahepatic or synchronous disease have the greatest survival benefit from surgery [63].

Two-stage procedures for synchronous bilobar liver metastases from NET, including portal vein embolization, enables complete resection and good long-term outcome in selected patients [68]. Debulking extends survival although recurrence is expected [69–71]. Surgical treatment of metastatic PNET should be performed in specialized centers and managed with a multidisciplinary approach [57, 72]. 

### 4.2. Technical Aspects

#### 4.2.1. Resection versus Enucleation

Standard surgical approaches to PNETs include pancreaticoduodenectomy and distal or subtotal pancreatectomy. Middle segment pancreatectomy is an alternative in the management of PNETs located in the neck or body of the pancreas [73]. A general risk of major pancreatic resections is functional impairment of the organ due to loss of parenchyma, resulting in exocrine and/or endocrine insufficiency. Thus, parenchyma-sparing surgical techniques should be attempted when possible. Enucleation is a feasible procedure for the radical treatment of benign and borderline pancreatic neoplasms [74] and is associated with long-term survival, despite a relatively high risk of pancreatic fistula formation [75, 76]. Before enucleating a PNET, it is important to consider where the tumor is located in relation to the main pancreatic duct, as enucleations of tumors located very close to this may result in damage to the pancreatic duct and subsequent pancreatic leakage. Decisions regarding enucleations are highly individual compared to standard resections, underlining the importance of treatment in experienced high-volume institutions. Tumor enucleation is associated with shorter operative time, less intraoperative blood loss, and shorter hospital stay compared to pancreaticoduodenectomy and distal pancreatectomy [74]. 

#### 4.2.2. Open versus Laparoscopic Surgery

Over the last decade there has been a trend towards more parenchyma-sparing and minimally invasive techniques in the management of PNETs. This shift has not increased morbidity or compromised survival [77]. Laparoscopic surgery for small and solitary PNETs is feasible and safe [78–81]. Advantages of the minimally invasive approach are less intraoperative bleeding [82], faster postoperative recovery [83], shorter hospital stay [84, 85], and improved cosmesis, compared to the open approach.

Laparoscopic distal pancreatectomy (LDP) is today an established procedure at several institutions worldwide [86–94]. The procedure provides similar short- and long-term oncologic outcomes as open distal pancreatectomy [85] and a selective use of it also seems to be a cost-efficient alternative to open distal pancreatectomy [82]. LDP with preservation of the spleen is feasible with a moderate risk of postoperative splenic infarction [95]. However, the significance of spleen preservation on oncologic outcome in patients with PNET remains unclear. Beside LDP of PNET in the pancreatic body and tail, laparoscopic enucleation of nonfunctioning PNETs in the pancreatic head [96] and laparoscopic pylorus-preserving pancreatoduodenectomy are feasible procedures that can be considered in selected cases [97]. When performing laparoscopic pancreatic surgery for PNET, intraoperative laparoscopic ultrasound should always be applied, as this allows safe tumor dissection and excision [80]. If the tumor cannot be identified precisely by laparoscopic ultrasound, conversion to open surgery should be considered [98]. Laparoscopic pancreatic surgery demands a high level of surgical skills in minimally invasive surgery and should be performed in specialized centers [99]. 

#### 4.2.3. Lymph Node Sampling

From studies performed on pancreatic ductal adenocarcinoma, it is known that lymph node status is an important prognostic factor in resectable disease [100–102]. This has also been demonstrated in studies on PNET, where lymph node ratio is a significant predictor of recurrence after curative resection for malignant PNETs [103], and lymph node metastases in PNETs are related to better survival [104]. In many surgical specimens of PNETs, lymph nodes are not evaluated by the pathologist [105]. This may result in understaging of patients with potentially inadequate resection. It is of great importance to know to what extent parenchyma-sparing and minimally invasive pancreatic surgery can provide sufficient lymph node sampling for optimal oncologic outcome. When compared to open surgery, there are studies concluding with a clear limitation of LDP [84] as well as studies concluding with a comparable lymph node sampling after LDP [63]. Enucleations are associated with a low lymph node sampling rate compared with standard resections [105]. Lymph node sampling should be performed routinely when performing parenchyma-preserving or minimally invasive removal for small PNETs, to avoid understaging [34, 43]. Moreover, frozen-section examination should be performed, and when malignancy is confirmed, oncologically appropriate lymph node dissection is recommended [43]. 

#### 4.2.4. Vascular Reconstruction

Surgery for locally advanced PNETs with vascular involvement is controversial. Vascular reconstruction has already been established in the treatment of locally advanced pancreatic adenocarcinoma [106]. Several case reports [107–109] suggest that a similar approach is feasible and beneficial in selected patients with PNETs. Norton et al. have recently examined this issue systematically [110]. In their study, only 9 of 42 patients with major vascular abutment undergoing resections of PNETs required vascular reconstruction. This shows that in most cases, even if the radiological evaluation suggests vascular involvement and at surgery the PNET is found to partially encase or involve the vessel, the tumor can be removed with careful dissection without requiring vascular reconstruction. Conventional contraindications to surgical resection of pancreatic malignancy, such as superior mesenteric vein invasion, should be reconsidered in patients with locally advanced PNETs [52, 110]. 

## 5. Prognosis and Follow-up

The five- and 10-year survival rates for all PNETs are about 65% and 45%, respectively, [19, 111]. The five-year survival rate for functioning PNETs is about 80% [111], while the five- and 10-year survival rates for nonfunctioning PNETs are about 55% and 30%, respectively, [11, 111]. Definitive surgical resection of the primary tumor, absence of liver metastases, metachronous liver metastases, and aggressive treatment of the liver metastases are predictive factors of long-term survival in patients with PNETs [112].

Long-term follow-up of patients having undergone surgical treatment for nonfunctioning PNETs is essential as there is a risk of late recurrence [34]. There have recently been published several international consensus guidelines on the management of patients with PNETs [113–116], which also include guidelines on follow-up of patients with functioning PNETs [113], nonfunctioning PNETs [114] and NECs [48]. The follow-up of patients with PNETs should be managed by specialized centers with a multidisciplinary approach [16, 57, 72, 117, 118]. 

## 6. Conclusions

In conclusion, this paper shows how sporadic PNETs represent a rare and clinically diverse group of pancreatic neoplasms, which requires special attention from hepatopancreatobiliary surgeons. Even though sporadic PNETs are associated with a high malignant potential, they are generally slow-growing. This explains why conventional contraindications to surgical resection of pancreatic malignancy should be reconsidered in patients with locally advanced or even metastatic disease. There is still a need for an internationally accepted histopathological classification system for PNETs. Recent studies suggest that the ENETS TNM staging system is a reliable system in terms of prognostic stratification. Other key points related to surgical treatment of sporadic PNETs with specific focus on surgical approaches and tumor classification are shown in Table 3. 

## Figures and Tables

**Table 1:** Tumor-node-metastasis definitions in the European Neuroendocrine Tumor Society (ENETS) for staging for pancreatic neuroendocrine tumors [14, 15].

T—primary tumor	
Tx	Primary tumor cannot be assessed
T0	No evidence of primary tumor
T1	Tumor limited to the pancreas and size <2 cm
T2	Tumor limited to the pancreas and size 2–4 cm
T3	Tumor limited to the pancreas and size >4 cm or invading duodenum or bile duct
T4	Tumor invading adjacent organs (stomach, spleen, colon, adrenal gland) or wall of large vessels (celiac axis or superior mesenteric artery)

N—regional lymph nodes	

Nx	Regional lymph node cannot be assessed
N0	No regional lymph node metastasis
N1	Regional lymph node metastasis

M—distant metastasis	

Mx	Distant metastasis cannot be assessed
M0	No distant metastasis
M1	Distant metastasis

**Table 2:** Pathology report recommendations for pancreatic neuroendocrine tumors (PNETs) [4].

Macroscopic description	
(i) Exact anatomical site	
(ii) Margins distance	
(iii) Size of the lesion	

Microscopic description	

(i) Description inclusive of all relevant aspects according to specific anatomical site (structure, necrosis, etc.)	
(ii) Supporting immunohistochemistry	
(iii) Mitotic count per 10 HPF (2 mm^2^) and number of mitoses assessed in 50 HPF	
(iv) Ki-67 index per 400–2,000 cells (hot spots)	
(v) Node status	
(vi) Margins status	

Diagnosis	

(i) Definition (NET or NEC)	
(ii) Cell component (functioning cases only)	
(iii) Grade (1, 2, or 3)	
(iv) Tumor-node-metastasis stage	

HPF: high-power field; NEC: neuroendocrine carcinoma; NET: neuroendocrine tumor.

**Table 3:** Key points.

Key points	
(i) Surgical resection is the only curative treatment for patients with PNETs.	
	
(ii) The incidence of PNETs has increased during the last decades.	
	
(iii) PNETs are clinically diverse and divided in functioning and nonfunctioning disease.	
	
(iv) The ENETS TNM staging system is superior to the UICC/AJCC/WHO 2010 TNM staging system in terms of prognostic stratification for patients with PNETs.	
	
(v) There has been a trend towards more aggressive surgery as well as more minimally invasive surgery in patients with PNETs over the last decade.	
	
(vi) Lymph node sampling should be performed routinely after curative resection of PNETs, as lymph node ratio is a significant predictor of recurrence.	
	
(vii) The five- and 10-year survival rates for all PNETs are about 65% and 45%.	
	
(viii) Long-term followup of patients having undergone surgical treatment for nonfunctioning PNETs is essential due to the risk of late recurrence.	
